# Pelvic Floor Distress and Sexual Function Across Self-Reported Primary Exercise Modalities in Physically Active Nulliparous Women

**DOI:** 10.3390/sports14070309

**Published:** 2026-07-21

**Authors:** Elizabeth Templeton, Susan Shirley, Daniela Lopez Berazain, Rob Sillevis

**Affiliations:** Department of Rehabilitation Sciences, Florida Gulf Coast University, Fort Myers, FL 33965, USA; etempleton@fgcu.edu (E.T.);

**Keywords:** pelvic floor dysfunction, nulliparous women, high-intensity functional training, sexual function

## Abstract

Background: High-intensity and high-impact exercise may increase intra-abdominal pressure and loading of the pelvic floor. Although pelvic floor dysfunction (PFD) has been reported among physically active women, findings remain inconsistent, particularly in young nulliparous women. This study examined the relationship between exercise modality, pelvic floor distress, and sexual function in physically active nulliparous women. Methods: A cross-sectional survey was completed by 103 physically active nulliparous women aged 18–30 years. Participants completed the Pelvic Floor Distress Inventory-20 (PFDI-20) and Female Sexual Function Index (FSFI) and reported their primary exercise modality, exercise frequency, and duration. Statistical analyses included Welch’s ANOVA with effect sizes (ω^2^) and Spearman correlation analyses. Results: Participants engaged in aerobic training (10%), yoga/Pilates (6%), strength training combined with cardiovascular exercise (57%), powerlifting (14%), and CrossFit (14%). No statistically significant differences were detected across exercise modalities for total PFDI-20 scores (*p* = 0.209, ω^2^ = 0.05) or total FSFI scores (*p* = 0.883, ω^2^ = 0.00). Likewise, no significant differences were observed across individual PFDI-20 or FSFI domains (all *p* > 0.05), with effect sizes ranging from negligible to small (ω^2^ = 0.00–0.07). Weak exploratory correlations were identified between pelvic floor distress and selected sexual function domains (ρ = 0.18–0.23, *p* < 0.05). Conclusions: No statistically significant differences in pelvic floor distress or sexual function were detected among self-reported exercise modalities in this cohort. These exploratory findings should be interpreted cautiously and warrant confirmation through adequately powered longitudinal studies incorporating objective assessments of pelvic floor function.

## 1. Introduction

Participation in structured exercise programs, particularly those involving high-intensity functional training, has increased substantially among women in recent decades [1]. Modalities such as CrossFit, powerlifting, and combined strength–cardiovascular training emphasize multi-joint movements performed under elevated physiological demand [2,3]. These training approaches provide well-established cardiovascular, metabolic, and musculoskeletal benefits, including improvements in strength, bone density, body composition, and physical performance. However, high-intensity functional training frequently involves elevated and/or prolonged intra-abdominal pressure (IAP), repetitive high-impact loading, and heavy resistance efforts, which may create unintended adverse effects on pelvic floor health, particularly among women [4]. As female participation in competitive and recreational strength-based exercise continues to rise, understanding the balance between performance benefits and potential pelvic health consequences has become increasingly important.

The pelvic floor is a complex neuromuscular system responsible for maintaining continence, supporting pelvic organs, and contributing to lumbopelvic stability. Physiologically, the pelvic floor functions as an integral component of the lumbopelvic cylinder, coordinating with the diaphragm, abdominal wall, and spinal stabilizers to regulate IAP and maintain continence [5]. During heavy lifting or high-impact activity (such as repetitive jumping), IAP may increase several-fold to stabilize the spine and effectively transmit axial force. When these pressure demands are frequent, prolonged, or poorly managed, excessive loading may occur across pelvic connective tissue, neural structures, and musculature, potentially contributing to pelvic floor dysfunction [6]. This concern is particularly relevant in women because strenuous physical activity can substantially increase intra-abdominal pressure, a proposed mechanism underlying pelvic floor dysfunction [7].

Pelvic floor dysfunction (PFD) encompasses a spectrum of conditions, including urinary incontinence (UI), pelvic organ prolapse (POP) and sexual dysfunction, all of which can substantially impair quality of life, exercise participation, and psychosocial well-being [8,9]. Sexual function represents an important but often overlooked component of pelvic health. The pelvic floor contributes to sexual arousal, lubrication, orgasm, and satisfaction through both muscular support and neurosensory mechanisms. Despite this, relatively few studies have examined sexual function in physically active populations, particularly in relation to exercise modality and training intensity.

Pelvic floor-related conditions are common yet frequently under-recognized among women across the lifespan. Estimates suggest that nearly one in four women experience some degree of PFD [10,11,12,13]. In contrast, current evidence suggests that pelvic floor-related symptoms may be even more common in females participating in high-impact functional-fitness sports, with prevalence estimates reported as high as 40% [14]. This elevated prevalence supports the concept of athletic incontinence, defined as transient leakage or pelvic distress triggered by sport-specific loading demands rather than chronic pathology [15,16]. It appears that exercise intensity, ground reaction forces, movement repetition, and cumulative training volume may collectively determine the degree of pelvic loading risk [16]. However, the threshold at which physical training transitions from beneficial adaptation to detrimental pelvic stress remains poorly defined.

Pelvic floor disorders represent a substantial public health concern and impose a significant economic burden on healthcare systems. The annual cost of managing pelvic floor disorders exceeds $82 billion [17,18]; however, these estimates reflect the overall population affected by pelvic floor disorders, including many women whose symptoms are associated with pregnancy, childbirth, and aging. Although these factors are well-established contributors to pelvic floor dysfunction, less is known about the potential influence of different exercise modalities in otherwise healthy, nulliparous women. Understanding this relationship may help identify modifiable risk factors and inform prevention, early detection, and targeted education for physically active women. Awareness and understanding of pelvic floor function remain limited among female athletes and even fitness professionals [19,20]. Many continue to use high-pressure breathing strategies, such as the Valsalva maneuver, without concurrent pelvic floor protection or pressure-modulation strategies [21]. As a result, some women may self-limit exercise intensity, withdraw from sport participation, or experience reductions in confidence and quality of life [22].

Emerging evidence suggests that pelvic floor training integrated into general strength and conditioning programs can significantly reduce the incidence and severity of incontinence symptoms without compromising athletic performance [23,24]. However, much of the available literature includes heterogeneous samples, often combining parous and nulliparous women. Because pregnancy and childbirth are well-established contributors to pelvic floor dysfunction, mixed-parity samples limit the ability to isolate the independent effects of exercise exposure. Accordingly, the study of nulliparous women provides a more controlled framework for evaluating the relationship between exercise modality and pelvic floor health.

Given the increasing popularity of high-intensity exercise and the limited data available in nulliparous women, there remains a need to better understand how different exercise modalities relate to pelvic floor symptoms and sexual function. The purpose of this study was to compare pelvic floor distress and sexual function across self-reported primary exercise modalities in physically active nulliparous women. It was hypothesized that higher-impact exercise modalities would be associated with increased pelvic floor distress and reduced sexual function compared to lower-impact activities.

## 2. Materials and Methods

### 2.1. Study Design and Participants

This study used a quantitative cross-sectional observational study design to examine the relationship between self-reported high-intensity exercise modalities and pelvic floor and sexual health outcomes. This study was designed as an exploratory cross-sectional survey. No a priori sample size calculation was performed because the final sample size was determined by the number of eligible participants who voluntarily responded during the recruitment period. Participants were recruited through a snowball sampling technique, with phone calls, emails, fliers, and social media used to spread awareness among the target population. To be eligible for this study, participants were required to be nulliparous individuals assigned female at birth, aged 18–30 years, who had participated in structured exercise at least twice per week for the preceding six months. Participants meeting the inclusion criteria were excluded if they were pregnant, had a history of childbirth, previous pelvic surgery, or a neurological or musculoskeletal condition known to affect pelvic floor or sexual function. In addition to determining eligibility, participants were asked to report the duration of participation in their current primary exercise modality (<3 months, 3–5 months, 6–12 months, or >12 months). This variable was collected to characterize participants’ experience with their current exercise modality and was independent of the eligibility criterion requiring at least six months of regular exercise. This study was approved by the Florida Gulf Coast University Institutional Review Board (Protocol ID # 2024-46, 24 April 2024). In accordance with the Declaration of Helsinki, all participants provided informed consent before inclusion and agreed that their study information would be published anonymously.

**Figure 1:**
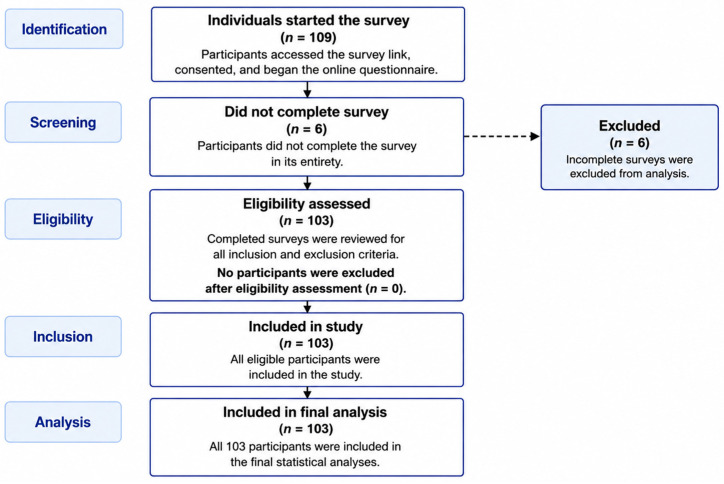
Flow diagram of participant recruitment, eligibility screening, and inclusion in the final analysis.

Participants completed an online Qualtrics^®^ survey (Provo, UT, USA), which included demographic information, exercise habits, and validated outcome measures. Exercise modality was self-reported. Participants were asked to identify the single exercise modality that best represented their usual form of physical activity and were categorized into one of five mutually exclusive groups: strength training combined with cardiovascular exercise, CrossFit, powerlifting, aerobic training, or yoga/Pilates. Participants selecting strength training combined with cardiovascular exercise reported a combination of structured resistance and cardiovascular training as their primary exercise modality, whereas participants classified in the aerobic exercise group reported predominantly aerobic forms of exercise. Classification was therefore based on participants’ self-selected primary exercise modality, and participation in multiple exercise modalities was not recorded. A total of 103 participants met the eligibility criteria and were included in the final analysis. A total of 109 participants initiated the survey. Eligibility was determined through an initial screening section of the survey. Participants who met the eligibility criteria were permitted to continue to the study questionnaire. Although one participant did not report their exact age in the demographic section, eligibility had already been confirmed during the screening process. Participants who initiated but did not complete the survey were excluded prior to eligibility assessment and data analysis. Eligibility was evaluated only for participants who completed the survey, and all 103 completed responses met the predefined inclusion and exclusion criteria. Consequently, analyses were performed using complete-case data from the 103 participants who completed all required study measures and met the eligibility criteria; no imputation of missing data was performed (Figure 1).

### 2.2. Outcome Measures

Pelvic floor distress was assessed using the Pelvic Floor Distress Inventory-20 (PFDI-20), a validated questionnaire that evaluates symptoms across three domains: pelvic organ prolapse, colorectal–anal distress, and urinary distress [25]. Originally developed by Barber et al. [26], the instrument has been extensively validated and demonstrates strong psychometric properties across diverse populations [26]. The PFDI-20 is used to measure the presence and severity of pelvic floor symptoms using three subscales: Pelvic Organ Prolapse Distress Inventory (6 questions-POPDI-6), Colorectal–Anal Distress Inventory (8 questions-CRADI-8), and Urinary Distress Inventory (6 questions-UDI-6). Each item is rated from 0 (“not at all”) to 4 (“quite a bit”) [27].

Each of the three PFDI-20 subscales is calculated by summing the responses within the domain, dividing by the number of completed items, and multiplying by 25, resulting in subscale scores ranging from 0 to 100. The total PFDI-20 score ranges from 0 to 300, with higher scores reflecting greater self-reported pelvic floor symptom distress [26]. The minimum possible score for each subscale is 0 and the maximum possible score is 100. Consistent with current recommendations, PFDI-20 scores are best interpreted using validated scoring approaches and longitudinal changes rather than arbitrary severity cutoffs [28]. The PFDI-20 has a sensitivity of 0.83 and a specificity of 0.79. The PFDI-20 exhibits excellent internal consistency, with Cronbach’s α values ranging from 0.71 to 0.89, and high test–retest reliability, with intraclass correlation coefficients (ICCs) typically ≥0.90 across both clinical and community-based cohorts [27]. Construct validity has been consistently demonstrated, with significantly higher PFDI-20 scores among women diagnosed with pelvic floor disorders compared with asymptomatic controls [29,30].

Sexual function was assessed using the Female Sexual Function Index (FSFI), a validated 19-item, multidimensional questionnaire instrument measuring six domains of sexual function, including desire, arousal, lubrication, orgasm, satisfaction, and pain [28]. Each item is scored either between 0 and 5 or 1 and 6. Scores are summed within each domain and multiplied by a domain factor ratio (0.6 for desire, 0.3 for arousal; 0.3 for lubrication; 0.4 for orgasm; 0.4 for satisfaction; and 0.4 for pain) in order to place all domain totals on a more comparable scale, and then subsequently summed to derive a total FSFI score.

The added domain scores determine the FSFI total score, which ranges from 2 to 36, with higher scores indicating better sexual function. Higher scores reflect better self-reported sexual function. In the present study, FSFI scores were used to compare sexual function across exercise modality groups and were not used to classify participants according to established clinical cutoff values [31]. It should be noted that sexual activity within the previous four weeks, a requirement for valid FSFI interpretation, was not verified in this study. The FSFI has a sensitivity of 0.96 and a specificity of 0.91 [32]. The FSFI was initially developed and validated by Rosen et al. [33], demonstrating high internal consistency (Cronbach’s α > 0.90 for total score and domains) and excellent test–retest reliability (r = 0.79–0.91 across subscales). Its comprehensive coverage of sexual function and clear psychometric strength have led to its recognition as the gold standard for assessing female sexual function in both clinical and research contexts.

Clinically, the FSFI’s construct validity is further supported by its moderate-to-strong correlations with measures of depression, anxiety, relationship satisfaction, and pelvic floor health [31]. These associations highlight the biopsychosocial interplay underlying sexual function and emphasize the tool’s multidimensional relevance in women’s health. Additionally, recent evidence indicates the FSFI can sensitively capture changes in sexual function associated with exercise, childbirth, and pelvic floor dysfunction, making it especially relevant for research involving the high-intensity athlete [34].

### 2.3. Statistical Analysis

Statistical analyses were performed using Jamovi^®^ software (Version 2.7). Descriptive statistics were calculated for all study variables and are presented as means and standard deviations (SD). Normality was assessed using the Shapiro–Wilk test, and homogeneity of variances was evaluated using Levene’s test. Because exercise groups were unequal in size and several outcomes demonstrated potential violations of the homogeneity of variance assumption, group differences were evaluated using Welch’s analysis of variance (ANOVA), which is robust to unequal variances and unequal sample sizes. Effect sizes were estimated using omega squared (ω^2^), a bias-corrected measure of explained variance that is recommended for ANOVA designs because it provides a less biased estimate of population effect size than eta squared (η^2^), particularly in studies with relatively small or unequal group sizes. Negative ω^2^ estimates, which may occur because ω^2^ is a bias-corrected estimator, were reported as 0.00, indicating negligible effect sizes. Spearman’s rank correlation coefficients were calculated to examine associations between pelvic floor distress and sexual function variables. Statistical significance was established at *p* < 0.05. Correlation analyses were considered secondary exploratory analyses intended to identify potential associations between questionnaire domains; therefore, these findings should be interpreted as hypothesis-generating rather than confirmatory.

## 3. Results

A total of 103 subjects completed the survey; however, only 102 provided age information. The age distribution was relatively balanced across early adulthood. Nearly half of the participants (49%) were aged 23 to 26, followed by 27% aged 27 to 30, and 24% aged 18 to 22 (Table 1). Participants reported a wide range of physical activity practices. The majority (57%) identified strength training combined with cardio as their primary exercise type, followed by powerlifting (14%) and CrossFit (14%). Smaller proportions engaged in aerobic exercise (10%) or yoga/Pilates (6%). Most participants reported frequent and sustained exercise habits. A majority (65%) exercised three to five times per week, with another 22% reporting more than five sessions per week. Only a small fraction (2%) exercised fewer than three times per week. The vast majority of the participants (80%) had been exercising regularly for over a year, while 20% had adopted regular activity more recently (within the past year). Most participants (62%) typically worked out for 60 min, followed by 30 min (16%) and 90 min (15%) sessions. A small subset (8%) reported typical workouts longer than 90 min.

Table 2 presents the mean PFDI-20 domain scores across the five exercise modality groups. Descriptive differences were observed between exercise groups, with the Yoga/Pilates group demonstrating the highest mean POPDI-6 and CRADI-8 scores and the CrossFit group demonstrating the highest mean UDI-6 score. However, these descriptive differences were not statistically significant.

Table 3 presents the mean values of the FSFI sexual functioning subscales by exercise group. A higher score (max 6) indicated better sexual functioning. The Yoga/Pilates exercise group scores the lowest (2.90) on the Desire sub-scale, followed by the CrossFit group (3.09). The Aerobic group scores lowest on the Arousal subscale (1.53), followed by the CrossFit group (1.86). The Aerobic group scores lowest on the Lubrication subscale (1.98), followed by the CrossFit group (2.55). The Aerobic group scores lowest on the Orgasm subscale (1.80), followed by the Yoga/Pilates group (2.07). The Yoga/Pilates exercise group scores lowest on the Satisfaction subscale (1.47), followed by the Aerobic group (2.00). The Aerobic exercise group scores lowest on the pain subscale (2.32), followed by the Powerlifting group (2.94).

Exploratory Spearman rank correlations were computed to examine associations between PFDI-20 subscales and FSFI domains (Table 4). As expected, strong positive correlations were observed among the FSFI domains and among the PFDI-20 subscales, reflecting the related constructs measured within each instrument. Weak positive correlations (ρ = 0.18–0.23) were identified between selected PFDI-20 subscales and the FSFI domains of arousal, lubrication, and orgasm. These correlations are reported using nominal (unadjusted) *p* values and should be interpreted cautiously given the exploratory nature of the analyses, the number of correlations examined, and the absence of adjustment for multiple comparisons. Although several correlations reached nominal statistical significance, their magnitude was small and should be considered hypothesis-generating rather than confirmatory. Overall, no clinically meaningful relationship between pelvic floor distress and sexual function was demonstrated in this sample.

The POPDI-6 scores had a low degree of correlation. However, they were significant for arousal (ρ = 0.23) and orgasm (ρ = 0.21), and a nonsignificant low degree correlation with lubrication (ρ = 0.12), suggesting that pelvic organ prolapse distress symptoms may modestly relate to sexual functioning. The CRADI-8 had a low degree of correlation but significant orgasm (ρ = 0.22), and a nonsignificant low degree of correlation with lubrication (ρ = 0.19) and arousal (ρ = 0.18), suggesting that colorectal–anal distress symptoms may modestly relate to sexual functioning. Lastly, the UDI-6 showed a low but significant correlation with arousal (ρ = 0.22) and lubrication (ρ = 0.21), and a nonsignificant, low degree of correlation with orgasm (ρ = 0.19), suggesting that urinary symptoms may modestly relate to sexual functioning.

Analysis of variance revealed no statistically significant differences across exercise modalities for total pelvic floor distress scores, as measured by the PFDI-20 (Welch’s F(4, 18.8) = 1.63, *p* = 0.209, ω^2^ = 0.05), or for total sexual function scores, as measured by the FSFI (Welch’s F(4, 20.4) = 0.29, *p* = 0.883, ω^2^ = 0.00). The observed effect size for the PFDI-20 total score was small, whereas the effect size for the FSFI total score was negligible. Similarly, no statistically significant differences were identified across exercise modalities for any PFDI-20 or FSFI subscale (all *p* > 0.05), with effect sizes ranging from negligible (ω^2^ = 0.00) to small (ω^2^ = 0.07). Group means, standard deviations, Welch’s ANOVA results, and effect sizes are presented in Table 5.

Although not statistically significant, descriptive trends were observed. Participants in the CrossFit group demonstrated slightly higher urinary distress scores, while some smaller subgroups exhibited lower scores in arousal and lubrication domains of the FSFI. However, these findings should be interpreted cautiously due to small sample sizes within these groups.

## 4. Discussion

The purpose of this study was to examine self-reported pelvic floor distress and sexual function in young, nulliparous women participating in a variety of self-reported exercise modalities, with the broader aim of exploring the relationship between exercise modality and pelvic floor health. By focusing exclusively on young, nulliparous women, this study sought to isolate the influence of exercise-related biomechanical loading from established risk factors such as childbirth, parity, and age-related connective tissue changes. Notably, age alone may not be determinative of pelvic floor weakness; rather, limited awareness of PFM function and inadequate conditioning may contribute to progressive decline over time [23,24].

The primary finding of the present study was that no statistically significant differences were observed across self-reported exercise modalities for either pelvic floor distress or sexual function outcomes. Contrary to our hypothesis, no statistically significant differences in pelvic floor distress or Female Sexual Function Index scores were detected among participants engaging in high-impact or high-intensity exercise modalities, including CrossFit, compared with those participating in aerobic, strength, or combined exercise programs. These findings indicate that no statistically significant differences in pelvic floor distress or FSFI scores were detected among exercise modalities within this sample. However, these findings should be interpreted cautiously. The relatively small and unequal group sizes may have limited the statistical power to detect small-to-moderate between-group differences. Furthermore, although the FSFI instructs participants to report their sexual thoughts, feelings, and experiences during the preceding four weeks, recent sexual activity was not independently verified. Consequently, the FSFI findings should be interpreted as comparative questionnaire scores between exercise modality groups rather than as indicators of normal or impaired sexual function or the prevalence of female sexual dysfunction.

Our findings differ from several previous studies reporting a higher prevalence of urinary symptoms among women participating in CrossFit and other high-impact functional fitness programs [14,15,16,35]. Prior investigations have identified increased urinary incontinence during activities such as jumping, running, and heavy lifting [36,37,38]. CrossFit has received particular attention because it incorporates movements that are thought to place substantial demands on the pelvic floor, including high-impact exercises (e.g., box jumps, double-unders, and running) and heavy resistance exercises (e.g., squats, deadlifts, and Olympic lifts), which are frequently performed using the Valsalva maneuver. These activities can generate substantial increases in intra-abdominal pressure and have therefore been proposed as potential contributors to pelvic floor dysfunction in susceptible individuals.

Several factors may explain why our findings differ from the existing literature. Many previous studies included heterogeneous populations consisting of both parous and nulliparous women, broad age ranges, or individuals with prior pelvic floor injury, making it difficult to isolate the independent effects of exercise exposure. Pregnancy and childbirth remain among the strongest risk factors for pelvic floor dysfunction and may account for many of the symptoms reported in previous cohorts. By specifically examining healthy, young, nulliparous women, the present study minimized these important confounding factors and provided insight into pelvic floor health before pregnancy-related physiological changes occur.

It is also important to recognize that the present study categorized participants according to their primary self-reported exercise modality and did not collect detailed information regarding specific exercises performed, training volume, lifting loads, breathing strategies, or exercise intensity within each modality. Consequently, it is not possible to determine whether particular movement patterns or training characteristics influenced pelvic floor outcomes. Future studies should incorporate more detailed assessments of exercise exposure to better understand how specific movements, training loads, and breathing mechanics influence pelvic floor function in physically active women.

One possible explanation for the absence of detectable between-group differences is the physiologic resilience of the pelvic floor in younger women. Participants in the present cohort were under 30 years of age, an age range in which connective tissue integrity, neuromuscular responsiveness, and recovery capacity are generally more favorable. It is therefore plausible that young, nulliparous women possess greater resilience to the repetitive increases in intra-abdominal pressure associated with strenuous exercise. However, this hypothesis was not directly evaluated in the present study and should be interpreted cautiously. Previous literature demonstrating age- and parity-related declines in pelvic floor muscle strength and connective tissue support provides a potential biological rationale for this interpretation [39,40].

Similarly, women who regularly engage in resistance training or functional fitness may develop neuromuscular adaptations that contribute to improved trunk stability, movement control, and pressure management during physically demanding activities [41,42]. Evidence from pelvic floor rehabilitation suggests that coordinated movement training involving pelvic floor muscle activation may improve continence-related outcomes, although the certainty of this evidence is low and it has primarily been demonstrated in women with urinary incontinence rather than healthy athletic populations [42]. Although these adaptations have been proposed in the literature, they were not directly assessed in the present study. Consequently, any potential influence on pelvic floor function remains speculative. These proposed mechanisms represent one possible hypothesis for the absence of detectable between-group differences observed in the present study; however, this interpretation requires confirmation through prospective studies incorporating objective assessments of pelvic floor muscle function, breathing mechanics, neuromuscular activation, and more comprehensive measures of exercise exposure [43].

Although no statistically significant differences were observed, descriptive trends suggested slightly higher urinary distress among participants engaged in higher-impact exercise modalities, particularly CrossFit. While these findings did not reach statistical significance, they are consistent with the theoretical understanding that repetitive impact forces, rapid force transmission, and elevated intra-abdominal pressure may increase loading demands on pelvic floor tissues [14,15,16,36,37,38]. These trends warrant continued attention and further investigation in larger, adequately powered cohorts with more balanced group distributions.

The present study also found no statistically significant differences in sexual function across exercise modalities. Overall FSFI scores were comparable across exercise modalities. Because recent sexual activity was not independently verified, these findings should be interpreted as comparisons of questionnaire scores rather than indicators of normal or impaired sexual function [32]. These findings indicate that no statistically significant differences in pelvic floor distress or FSFI scores were detected among the exercise modalities evaluated in this sample. However, these results should not be interpreted as evidence that high-intensity exercise has no effect on pelvic floor or sexual health, as the study may have been underpowered to detect small-to-moderate differences and did not comprehensively characterize exercise exposure.

Weak but statistically significant correlations between pelvic floor distress subscales and selected sexual function domains were observed, indicating a modest relationship between pelvic symptoms and sexual well-being. Although these exploratory findings are consistent with previous literature suggesting that urinary symptoms, pelvic discomfort, body confidence, and fear of leakage may influence arousal, satisfaction, and sexual quality of life [7,43], the observed associations were weak and should not be interpreted as evidence of a clinically meaningful relationship. Pelvic health and sexual function likely exist within a broader biopsychosocial framework, influenced not only by physical symptoms but also by confidence, emotional well-being, and interpersonal factors [44,45].

It is important to interpret these exploratory correlation findings with caution. The observed associations were weak in magnitude and derived from multiple secondary correlation analyses, increasing the potential for Type I error. Consequently, these findings should be considered hypothesis-generating rather than confirmatory and warrant validation in larger, adequately powered studies using more comprehensive assessments of pelvic floor function and sexual health.

Clinically, these findings underscore the importance of screening for pelvic floor symptoms across exercise populations, including young nulliparous women who may otherwise be perceived as low risk. Pelvic floor symptoms are frequently normalized within athletic culture, particularly urinary leakage during high-intensity exercise, and therefore may go underreported [46]. Early recognition and education are essential to prevent progression into chronic dysfunction or exercise avoidance. Exercise professionals and rehabilitation providers should consider routine use of validated outcome measures such as the PFDI-20, with particular attention to urinary symptoms assessed through the UDI-6 subscale. In addition, coaching strategies that reinforce breath-pressure management, minimize unnecessary Valsalva during submaximal tasks, and progressively expose individuals to impact-based activity may be beneficial. Pelvic floor muscle training remains a first-line conservative intervention for women presenting with urinary symptoms and may be integrated proactively within athletic populations [47].

### 4.1. Limitations

Several limitations should be considered when interpreting these findings. First, this exploratory cross-sectional study did not include an a priori sample size calculation, and the relatively small sample size, particularly within the yoga/Pilates, aerobic, and CrossFit subgroups, may have limited the statistical power to detect subtle but clinically meaningful between-group differences. Recruitment through snowball sampling may also have favored health-conscious or asymptomatic participants, potentially biasing the sample toward healthier pelvic floor profiles.

Second, exercise exposure was characterized solely by participants’ self-reported primary exercise modality. Although participants were classified according to the exercise modality that best represented their usual form of physical activity, the survey did not capture participation in multiple exercise modalities or important training characteristics, including exercise intensity, weekly training volume, years of participation, competitive level, lifting loads, breathing strategies, Valsalva use, jumping or running volume, prior pelvic floor education, or participation in individual versus team sports. Consequently, exercise modality may not fully reflect the mechanical loading experienced by the pelvic floor and may have introduced heterogeneity within the exercise groups.

Third, the study relied on validated self-report questionnaires, which depend on participant awareness, interpretation, and willingness to disclose sensitive information, thereby introducing the potential for reporting bias. Additionally, several potentially important confounding variables were not collected or controlled for, including body mass index, hormonal contraceptive use, menstrual cycle phase, urinary tract history, relationship status, psychological distress, prior pelvic floor muscle training, and other factors that may influence pelvic floor symptoms or sexual function. Although the PFDI-20 provides a validated assessment of current self-reported pelvic floor symptom distress, it does not capture a comprehensive medical history or previous diagnoses of pelvic floor dysfunction. Furthermore, although the FSFI instructs participants to respond based on their sexual thoughts, feelings, and experiences during the preceding four weeks, recent sexual activity was not independently verified. Consequently, interpretation of absolute FSFI scores and established clinical cutoff values should be made with caution, particularly for participants reporting limited or no recent sexual activity.

Finally, the cross-sectional design precludes causal inference and does not permit conclusions regarding whether long-term participation in specific exercise modalities contributes to pelvic floor dysfunction, resilience, or both over time.

### 4.2. Future Directions

Future research should expand to larger and more diverse athletic cohorts and incorporate objective pelvic floor assessment methods alongside validated patient-reported outcome measures to provide a more comprehensive evaluation of pelvic floor health. Objective measures may include pelvic floor muscle strength assessed by digital examination or dynamometry, ultrasound imaging, electromyography, vaginal manometry, or pressure biofeedback. Prospective studies should examine how training duration, cumulative load exposure, breathing mechanics, lifting strategies, and pelvic floor awareness influence symptom development. Investigating whether early education and preventive pelvic floor muscle training can reduce symptom onset in female athletes may have substantial clinical and public health value. Longitudinal designs following women from their pre-childbearing years through pregnancy and the postpartum period would be particularly valuable in determining whether long-term exercise exposure contributes to pelvic floor resilience or vulnerability.

## 5. Conclusions

In this exploratory cross-sectional study, no statistically significant differences in pelvic floor distress or Female Sexual Function Index scores were detected across self-reported exercise modalities in physically active, nulliparous women. These findings should be interpreted cautiously given the relatively small and unequal group sizes and other methodological limitations. Nevertheless, this study contributes to the limited literature by examining pelvic floor health in young nulliparous women while minimizing the confounding effects of pregnancy and childbirth. Larger, adequately powered longitudinal studies incorporating objective assessments of pelvic floor function and more comprehensive measures of exercise exposure are needed to further clarify these relationships.

## Figures and Tables

**Table 1:** Participant Demographics and Exercise Characteristics. *N* = number of subjects, mo = months, min = minutes.

Characteristic	Category	*N*	%
Age			
	18–22	24	23.5%
	23–26	50	49.0%
	27–30	28	27.5%
Exercise Type			
	Aerobic	10	9.7%
	Yoga/Pilates	6	5.8%
	Strength + Cardio	59	57.3%
	Powerlifting	14	13.6%
	CrossFit	14	13.6%
Workouts per Week (days)			
	2–3	13	12.6%
	4–5	67	65.0%
	>5	23	22.3%
Duration of Current Primary Exercise Modality			
	<3 mo	2	1.9%
	3–5 mo	8	7.8%
	6–12 mo	11	10.7%
	>12 mo	82	79.6%
Typical Workout Length			
	30 min	16	15.5%
	60 min	64	62.1%
	90 min	15	14.6%
	>90 min	8	7.8%

**Table 2:** PFDI-20 scores among the three domains. *N* = number of subjects.

Exercise Type	*N*	POPDI-6	CRADI-8	UDI-6
Aerobic	10	12.50 ± 14.43	9.69 ± 10.15	17.08 ± 20.27
Yoga/Pilates	6	18.75 ± 16.40	22.92 ± 24.66	15.28 ± 19.84
Strength + Cardio	59	5.51 ± 7.52	9.22 ± 9.77	9.89 ± 14.26
Powerlifting	14	13.69 ± 20.11	10.49 ± 15.39	13.99 ± 18.39
CrossFit	14	12.50 ± 16.59	12.72 ± 11.20	16.96 ± 20.44

**Table 3:** FSFI scores for each exercise group per domain. *N* = number of subjects.

Exercise Type	*N*	Desire	Arousal	Lubrication	Orgasm	Satisfaction	Pain	FSFI Total
Aerobic	10	3.42 ± 1.96	1.53 ± 1.83	1.98 ± 2.09	1.80 ± 1.97	2.00 ± 1.81	2.32 ± 2.65	13.05 ± 10.52
Yoga/Pilates	6	2.90 ± 2.38	2.30 ± 2.08	2.60 ± 2.01	2.07 ± 1.69	1.47 ± 1.40	3.13 ± 2.67	14.47 ± 11.73
Strength + Cardio	59	3.40 ± 1.71	2.20 ± 1.66	2.79 ± 1.70	2.62 ± 1.64	2.03 ± 1.25	3.49 ± 2.30	16.53 ± 8.71
Powerlifting	14	3.56 ± 1.48	2.21 ± 1.62	2.98 ± 1.64	2.57 ± 1.51	2.11 ± 1.03	2.94 ± 2.48	16.37 ± 6.64
CrossFit	14	3.09 ± 1.69	1.86 ± 1.70	2.55 ± 1.99	2.49 ± 1.98	2.14 ± 1.45	3.09 ± 2.43	15.21 ± 8.62

**Table 4:** Exploratory Spearman rank correlation coefficients (ρ) between PFDI-20 subscales and FSFI domains. POPDI-6, Pelvic Organ Prolapse Distress Inventory; CRADI-8, Colorectal–Anal Distress Inventory; UDI-6, Urinary Distress Inventory. Values represent Spearman’s rank correlation coefficients (ρ). Nominal (unadjusted) *p* < 0.05. Correlation analyses were exploratory and hypothesis-generating; *p* values were not adjusted for multiple comparisons.

Scale	POPDI-6	CRADI-8	UDI-6	Desire	Arousal	Lubrication	Orgasm	Satisfaction
POPDI-6	-							
CRADI-8	0.51 *	-						
UDI-6	0.49 *	0.37 *	-					
Desire	0.01	0	0.04	-				
Arousal	0.23 *	0.18	0.22 *	0.52 *	-			
Lubrication	0.12	0.19	0.21 *	0.28 *	0.75 *	-		
Orgasm	0.21 *	0.22 *	0.19	0.33 *	0.80 *	0.75 *	-	
Satisfaction	0.14	0.07	0.04	0.48 *	0.59 *	0.47 *	0.56 *	-
Pain	0.01	0.12	0.12	0.16	0.55 *	0.66 *	0.57 *	0.21 *

Note: * Values represent Spearman’s rank correlation coefficients (ρ). Nominal (unadjusted) *p* < 0.05. Correlation analyses were exploratory and hypothesis-generating; *p* values were not adjusted for multiple comparisons.

**Table 5:** One-Way Welch’s ANOVA Comparing Group Differences Across PFDI-20 and FSFI Scores. F = Welch’s analysis of variance statistic; df_1_ = numerator degrees of freedom; df_2_ = denominator degrees of freedom. No statistically significant differences were observed across groups (all *p* > 0.05).

Scale	Aerobic (*n* = 10)	Yoga/Pilates (*n* = 6)	Strength + Cardio (*n* = 59)	Powerlifting (*n* = 14)	CrossFit (*n* = 14)	Welch’s *F* (df_1_*,* df_2_)	*p*	ω^2^
POPDI-6	12.50 ± 14.43	18.75 ± 16.40	5.51 ± 7.52	13.69 ± 20.11	12.50 ± 16.59	2.189 (4, 18.2)	0.111	0.07
CRADI-8	9.69 ± 10.15	22.92 ± 24.66	9.22 ± 9.77	10.49 ± 15.39	12.72 ± 11.20	0.652 (4, 19.4)	0.633	0.03
UDI-6	17.08 ± 20.27	15.28 ± 19.84	9.89 ± 14.26	13.99 ± 18.39	16.96 ± 20.44	0.677 (4, 19.4)	0.616	0.00
Desire	3.42 ± 1.96	2.90 ± 2.38	3.40 ± 1.71	3.56 ± 1.48	3.09 ± 1.69	0.199 (4, 20.3)	0.936	0.00
Arousal	1.53 ± 1.83	2.30 ± 2.08	2.20 ± 1.66	2.21 ± 1.62	1.86 ± 1.70	0.354 (4, 20.3)	0.838	0.00
Lubrication	1.98 ± 2.09	2.60 ± 2.01	2.79 ± 1.70	2.98 ± 1.64	2.55 ± 1.99	0.415 (4, 20.1)	0.795	0.00
Orgasm	1.80 ± 1.97	2.07 ± 1.69	2.62 ± 1.64	2.57 ± 1.51	2.49 ± 1.98	0.453 (4, 20.4)	0.769	0.00
Satisfaction	2.00 ± 1.81	1.47 ± 1.40	2.03 ± 1.25	2.11 ± 1.03	2.14 ± 1.45	0.262 (4, 20.3)	0.899	0.00
Pain	2.32 ± 2.65	3.13 ± 2.67	3.49 ± 2.30	2.94 ± 2.48	3.09 ± 2.43	0.493 (4, 20.2)	0.741	0.00
FSFI Total	13.05 ± 10.52	14.47 ± 11.73	16.53 ± 8.71	16.37 ± 6.64	15.21 ± 8.62	0.288 (4, 20.4)	0.883	0.00
PFDI Total	39.27 ± 38.52	56.94 ± 43.38	24.61 ± 23.99	38.17 ± 47.02	42.19 ± 39.01	1.629 (4, 18.8)	0.209	0.05

Note. F = Welch’s analysis of variance statistic; df_1_ = numerator degrees of freedom; df_2_ = denominator degrees of freedom. No statistically significant differences were observed across groups (all *p* > 0.05). Values are presented as mean ± standard deviation (SD). Group comparisons were performed using Welch’s ANOVA because of unequal sample sizes and potential heterogeneity of variances. Omega squared (ω^2^) values were estimated from the corresponding one-way ANOVA model to quantify effect size. Negative ω^2^ estimates, which may occur because ω^2^ is a bias-corrected estimator, were reported as 0.00, indicating negligible effect sizes.

## Data Availability

The original contributions presented in the study are included in the article; a de-identified dataset and analysis code are available from the corresponding author upon reasonable request.

## Abbreviations

The following abbreviations are used in this manuscript:

HIFT	High-intensity functional training
PFD	Pelvic floor dysfunction
PFDI-20	Pelvic Floor Distress Inventory-20
FSFI	Female Sexual Function Index
IAP	intra-abdominal pressure
UI	urinary incontinence
POP	pelvic organ prolapse
POPDI-6	Organ Prolapse Distress Inventory
CRADI-8	Colorectal–Anal Distress Inventory
UDI-6	Urinary Distress Inventory
